# Neuroimaging biomarkers of cognitive recovery after ischemic stroke

**DOI:** 10.3389/fneur.2022.923942

**Published:** 2022-12-14

**Authors:** Mouna Tahmi, Veronica A. Kane, Marykay A. Pavol, Imama A. Naqvi

**Affiliations:** ^1^Department of Neurology, State University of New York Downstate Health Sciences University, New York, NY, United States; ^2^Department of Medicine, Vagelos College of Physicians and Surgeons, Columbia University Irving Medical Center, New York, NY, United States; ^3^Department of Neurology and Rehabilitation and Regenerative Medicine, Columbia University, New York, NY, United States; ^4^Division of Stroke and Cerebrovascular Diseases, Department of Neurology, Columbia University, New York, NY, United States

**Keywords:** ischemic stroke, reperfusion, recovery, reorganization, neuroplasticity, neuroimaging, cognition

## Abstract

Post-stroke cognitive impairment affects more than one-third of patients after an ischemic stroke (IS). Identifying markers of potential cognitive recovery after ischemic stroke can guide patients' selection for treatments, enrollment in clinical trials, and cognitive rehabilitation methods to restore cognitive abilities in post-stroke patients. Despite the burden of post-stroke cognitive impairment, biomarkers of cognitive recovery are an understudied area of research. This narrative review summarizes and critically reviews the current literature on the use and utility of neuroimaging as a predictive biomarker of cognitive recovery after IS. Most studies included in this review utilized structural Magnetic Resonance Imaging (MRI) to predict cognitive recovery after IS; these studies highlighted baseline markers of cerebral small vessel disease and cortical atrophy as predictors of cognitive recovery. Functional Magnetic Resonance Imaging (fMRI) using resting-state functional connectivity and Diffusion Imaging are potential biomarkers of cognitive recovery after IS, although more precise predictive tools are needed. Comparison of these studies is limited by heterogeneity in cognitive assessments. For all modalities, current findings need replication in larger samples. Although no neuroimaging tool is ready for use as a biomarker at this stage, these studies suggest a clinically meaningful role for neuroimaging in predicting post-stroke cognitive recovery.

## Introduction

The American Heart Association (AHA) and the American Stroke Association (ASA) define ischemic stroke (IS) as an episode of neurological dysfunction caused by focal cerebral, spinal, or retinal infarction (1). IS represents 80% of all stroke types (2) and is a major cause of disability (3). With increasing survival after stroke and population aging, the prevalence of stroke is projected to increase by 3.4 million in 2030 (4, 5). Many patients who survive a stroke live with a significant long-term disability that affects multiple functions, including motor, sensory, language, and cognitive abilities. An increasing number of studies have attempted to determine potential factors that can influence recovery after stroke (6). In a pivotal report from the Stroke Rehabilitation Roundtable, the importance of using a biomarker approach to identify the potential for recovery after stroke was outlined (6). The consensus introduced the term Stroke Recovery Biomarker (SRB), defined as “indicators of disease state that can be used clinically as a measure reflecting underlying processes that may be difficult to measure directly in humans and could be used to predict recovery or treatment response” (6, 7). The report referred to recovery for several stroke-type deficits, including motor, sensory, language, and cognition. The SRB approach aims to guide patients' treatment selection, enrollment in clinical trials, and rehabilitation interventions (6, 7).

IS recovery studies have focused mostly on motor recovery (8–20). Cognition is another important domain frequently affected by IS, resulting in post-stroke cognitive impairment (PSCI). A recent systematic review and meta-analysis identified a pooled prevalence of PSCI of 39%, measured within the first year post-stroke (21). Others report a PSCI prevalence ranging from 20 to 80% depending on factors such as race and methodology (22). Cognitive recovery remains an understudied aspect of stroke, and no biomarkers are currently ready for use in clinical trials (6, 23). Some studies reported spontaneous restoration of cognitive function after the subacute phase of IS (24–26). However, many patients have cognitive impairment beyond the subacute phase of IS. A recent, large, population-based study of first-ever stroke patients from the South London Stroke Register between 1995 and 2018 (*n* = 6,504, mean age = 73 years) found that one-third of patients cognitively improved during the first 3 months post-stroke, one-third deteriorated, and the rest remained cognitively unchanged (27). The study further reported that PSCI was associated with a 5-year increase in the risk of mortality (RR = 30%), dependency (RR = 90%), depression (RR = 60%) and institutionalization (RR = 50%) (27).

Imaging is a potential biomarker for cognitive recovery after IS (6). A systematic review evaluating all biological and imaging markers found that global atrophy and medial temporal lobe atrophy were the most consistent predictors of cognitive impairment after stroke; however, this review did not link cognitive recovery over time with neuroimaging (28). Given the accessibility and the wide use of neuroimaging as part of stroke workup, neuroimaging is a promising tool to study the potential for cognitive recovery after stroke. Neuroimaging techniques are currently being used to understand higher cortical function and recovery among comatose patients with the eventual goal to identify potential early and tailored rehabilitative interventions and underlying patient-specific characteristics that are most responsive to these interventions (29, 30). Cognitive aging is another area where neuroimaging is increasingly used to comprehend brain cognitive processes (31). Thus, our goal for this review was to summarize literature within the last 10 years describing neuroimaging as a predictive marker of cognitive recovery in IS. We focus on IS, the most common type of stroke associated with PSCI. We highlight important findings and limitations in the studies and discuss some of the challenges for future studies to consider.

## Search methodology and literature selection

PubMed was used as the primary database for studies published in the last 10 years through September 6th, 2022. We used the Medical Subject Headings (MeSH) term “stroke” with the MeSH subheadings “complications” or “psychology” or the term “ischemic stroke” paired with both of the following terms in the abstract/title of each article or as MeSH terms:

- A cognition term (“cognition,” “cognitive,” “cognitive decline,” “cognition disorder,” or “dementia,” “neuropsychological,” or “neuropsychological tests”).
- A neuroimaging term (“neuroimaging,” “magnetic resonance imaging/MRI,” “functional magnetic resonance imaging/fMRI,” “diffusion tensor imaging/DTI,” “default mode network,” or “connectivity”).

Additional studies obtained through review of relevant article citations were included.

Studies examining other types of strokes—hemorrhagic stroke (HS), traumatic stroke, subarachnoid hemorrhage (SH), and transient ischemic attacks (TIA)—were excluded. HS were excluded due to their different recovery trajectories compared to IS (32, 33). Likewise, TIA were also excluded due to a lack of clear and persistent ischemic injury, which may result in a different recovery course (34, 35). Since cognitive recovery implies a change in cognitive performance over time, studies that reported only one cognitive assessment were excluded. Included studies associated a change in a cognitive assessment measure between at least two time points with baseline neuroimaging.

Finally, we required baseline imaging and cognitive assessments to be completed within 6 weeks of stroke. This is to ensure clinical relevance, as most imaging used to predict recovery would be completed during a hospital admission. A baseline cognitive assessment more than 6 weeks after an initial ischemic stroke may represent a different stage of stroke recovery and therefore not comparable to the other articles in this review.

**Figure 1:**
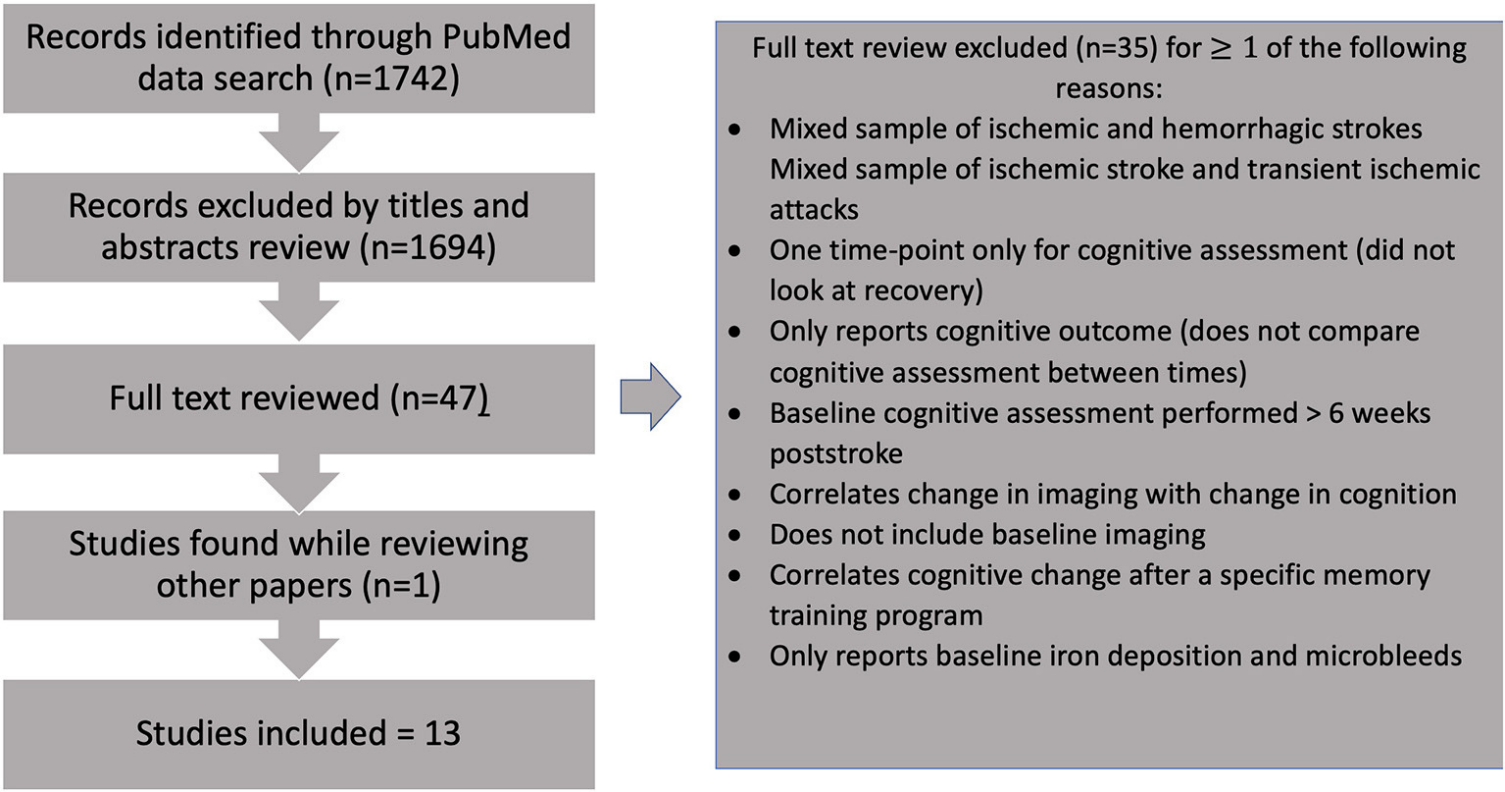
Flow diagram of study selection process.

Studies of potential relevance were selected, and 35 were excluded after careful full-text review based on the criteria detailed above (Figure 1, Supplementary Table 1). A total of 13 studies were included.

## Neuroimaging modalities used as biomarkers for cognitive recovery after ischemic stroke

### MRI: T1-weighted MRI, T2-weighted, and fluid-attenuated inversion recovery (FLAIR)

MRI as a means of characterizing or predicting cognitive impairment through structural biomarkers is well-documented (22, 36). These biomarkers include cerebral small vessel disease (SVD) such as white matter hyperintensities (WMH) and microinfarcts, cortical volume, and size and location of IS lesions (36). Researchers have attempted to use these same biomarkers to predict cognitive recovery following IS (37–39). Studies utilizing structural MRI that fit the criteria for this review are summarized in Table 1.

**Table 1:** Summary of key studies within the last 10 years on neuroimaging biomarkers of cognitive recovery after ischemic stroke.

**References**	**Study population, *N*, type of stroke, age**	**Duration of study**	**Type of imaging and imaging outcomes examined**	**Cognitive measures**	**Findings**
**fMRI**					
Vicentini et al. (40)	First subacute IS patients with no previous known neurologic disorder. Time 1 (*n* = 37, mean age = 62.92 ± 9.49 years, stroke onset = 24.32 ± 7.44 days, NIHSS: 2.66 ± 3.45). Time 2 after 6 months (*n* = 20, stroke onset: 182.05 ± 8.17 days). Cognitively healthy controls (*n* = 20)	6 months: 2-time points. fMRI and cognitive assessments were done at time 1 (subacute phase, within the first month) and 2 (chronic phase, after 6 months).	rsFC Three networks were examined: DMN, SN, and CEN network.	MoCA	At time 1 Patients had weaker interhemispheric connectivity in the DMN than controls (*p* = 0.028, FDR-corrected). Better cognitive performance was associated with a stronger interhemispheric (*r* = 0.409, FDR *p* = 0.058) and ipsilesional DMN connectivity (*r* = 449, FDR *p* = 0.068) and weaker contralesional SN connectivity (*r* = −0.426, FDR *p* = 0.049) At time 2 No change in functional connectivity in patients compared to time 1. Better cognitive recovery at time 2 was associated with stronger DMN connectivity (*r* = 0.511, FDR *p* = 0.090) and weaker SN interhemispheric subacute connectivity (*r* = −0.638, FDR *p* = 0.076)
**MRI: T1-weighted MRI, T2-weighted MRI, FLAIR**					
Fruhwirth et al. (41)	Recent small subcortical infarct (RSSI) patients (≤ 25 mm), no preexisting cognitive impairment (*N* = 82, mean age = 61 ± 10 years, 23% female)	15 months: Baseline cognitive assessment at time 1 (mean 6 days post-stroke) and time 2 (15 months). MRI at Time 1.	WMH lesion volume.	MoCA, Symbol Digital Modalities Test, Comprehensive Trail Making Test	All patients improved on MoCA, SDMT (processing speed) after 15 months, regardless of WMH severity (*p* = 0.011 for MoCA, *p* = 0.010 for SDMT (processing speed); after age adjustment, no difference between WMH severity and MoCA (*p* = 0.109) or SDMT (processing speed) (*p* = 0.414). No differences between CTMT-2 (attention) scores and WMH.
					CTMT-5 (set-shifting): no improvement for patients with mild (*p* = 0.086) or moderate-severe WMH (*p* = 0.801) at 15 months, compared to improvement in patients without WMH (*p* = 0.001). Same results even when corrected for age. WMH volume at baseline was only significant factor that predicted attention at 15 month follow up (*p* = 0.002) beyond demographics.
Scharf et al. (42)	Acute first-ever ischemic thalamic stroke, >18 yo Exclusion: previous psychiatric/neuro disorders (*N* = 37 case, 37 controls)	2 years: Neuropsychological assessment at time 1 (1 month), time 2 (6 month), time 3 (12 month) and time 4 (24 month) MRI at time 1, time 2, and time 4.	MRI—T1, T2, FLAIR, DWI	Digit span, rivermead behavioral memory test, Regensburg semantic and phonemic word fluency, TMA A/B, Stroop	Paramedian thalamic stroke patients demonstrated moderate language and executive deficits, with the best recovery of the three thalamic stroke topographies. Anterior thalamic stroke patients demonstrated the most severe deficits in verbal memory, language, and executive functions, which poorly recovered during follow-up. Inferolateral stroke patients also suffered from verbal memory, language, and executive deficits; the verbal memory and executive deficits recovered during follow-up, while the language deficits persisted.
Sagnier et al. (38)	Acute Ischemic supratentorial stroke patients with no previous neuropsychiatric disorder or dementia (*n* = 199, mean age = 65 ± 13, NIHSS median = 3 (4)	1 year: cognitive assessment was done at time 1 (baseline), time 2 (after 3 months), and time 3 (after 1 year). MRI was done at time 1 only (baseline between 24 and 72 h).	WMH, deep and lobar microbleeds, enlarged perivascular spaces in basal ganglia and centrum semiovale, previous small deep infarcts, and cSS.	MoCA ZCT processing speed and attention IST of verbal fluency for executive function.	- Cognitive performance improved more significantly in the first 3 months. - Severe WMH was identified in 34% of the patients, and cSS in 3.5%. Patients with severe WMH and focal cSS had overall worse cognitive performances.
					- Patient with severe WMH had less improvement over time in IST of verbal fluency (β = −0.16, *p* = 0.02) and the number of errors to ZCT (β = 0.19, *p* = 0.02). Those with focal cSS had less improvement over time for ZCT completion time (β = 0.14, *p* = 0.01) and number of errors (β = 0.17, *p* = 0.008).
Sagnier et al. (39)	Acute Ischemic supratentorial stroke patients with no previous neuropsychiatric disorder or dementia (*n* = 199, mean age = 67 ± 14, NIHSS ≥1)	One year: 3-time points cognitive assessment was done at time 1 (baseline), time 2 (after 3 months), and time 3 (after 1 year). MRI was done at time 1 only (baseline between 24 and 72 h)	CMI.	MoCA The Zazzo's cancellation task (ZCT) for processing speed and attention Issac set a test for verbal fluency (IST)	The number of CMI was associated with increased time at the ZCT over 1 year regardless of the other MRI markers, stroke severity, and demographic factors (B = 3.84, *P* = 0.01).
Sagnier et al. (43)	Acute supratentorial ischemic stroke without prestroke disability related to neurological disorder, mRS > 1 at baseline (*N* = 248, mean age 65 ± SD 14 years old, 66% men)	12 months: Baseline MRI Cognitive assessment at time 1 (within 24–72 h), time 2 (after 3 months), and time 3 (after 12 months)	WMH + stroke volume, gray matter (GM), white matter, and CSF volume.	Cognitive assessment: MoCA, Isaacs Set Test (IST), Zazzo's cancellation test Cognitive improvement, stability, or decline calculated using all 3 time points	- Radiographic model only: total GM volume was the only variable predictive of changes in all cognitive scores over the year of follow-up (*P* < 0.001 for MoCA, *P* = 0.03 for IST, *P* = 0.002 for time to perform Zazzo's cancellation task and *P* < 0.001 for the number of errors) - In clinical/radiographic model: total GM volume independently associated with cognition [MoCA (*P* = 0.04) and Zazzo's cancellation task (*P* = 0.04)] - GM volume of left fronto-temporo-insular regions, right temporo-insular cortex, and basal ganglia was significantly associated with cognitive improvement.
Sung et al. (44)	First ever ischemic stroke without specific etiologies predisposing to recurrence, cognitive impairment at baseline, neurodegenerative disease (*n* = 112, median age 64.5 (IQR 57.0–73.5) years, NIHSS at baseline 3.77 (IQR: 1.75–5).	1 year: MRI and cognitive assessment at time 1 (within 7 days), cognitive assessment again at time 2 (3 months) and time 3 (1 year)	Stroke location, SVD burden and hippocampal atrophy (HA) Modified cerebral small vessel disease (mCSVD) score calculated using lacunar infarction, microbleeds, moderate to severe perivascular space at the ganglionic level or a deep white matter Fazekas score ≥ 2. Medial temporal atrophy score used to determine hippocampal atrophy.	MoCA Weschler Adult Intelligence Scale III, Wechsler Memory Scale III the Semantic Association of Verbal Fluency Test for semantic verbal fluency, Wisconsin Card Sorting Test	No significant difference in change in MOCA scores between higher CSVD burden or abnormal HA. In the multivariate model, higher mCSVD score (adjusted odds ratio (aOR) 2.74, 95%CI 1.09–6.86, *p* = 0.032) independently predicted low cognitive performance at 1 year [but not an abnormal MTA score (aOR 1.53, 95%CI 0.56–4.21, *p* = 0.405)]. A combination of a higher mCSVD score and an abnormal MTA score resulted in the highest probability of classification in the LP group (aOR 4.18, 95%CI 1.05–16.66, *p* = 0.043).
Turunen et al. (45)	First ever supratentorial ischemic stroke, no baseline neurological or psychiatric disorder (*n* = 132, mean age 54 years, 68.2% male)	6 months: Baseline imaging Cognitive assessment at time 1 (~8 days post-stroke) and time 2 (6 month follow up)	Stroke location, categorized into two groups: infarction in cortical gray matter (including additional white matter) or infarction in subcortical gray and/or white matter.	Weschler memory scale, phonemic fluency task, TMA A/B, WMS-R, searching task On 6-month repeat, general intellect added	No differences in the recovery of cognitive profile amongst lesion location groups were found; after adjusting for baseline scores, the lesion location groups did not differ at follow-up.
Zhang et al. (46)	Imaging confirmed first time acute ischemic stroke, no history of cognitive or psychiatric disorder (*n* = 865, mean age 59.67 ± 10.92 years and 74.22% male)	12 months: Baseline imaging Baseline MOCA at time 1 (2 weeks/discharge) and time 2 (12 month follow-up)	Infarct location, small vessel disease features, WMHs, lacunes, microbleeds, enlarged perivascular spaces, cortical atrophy	MOCA Cognitive decline defined as reduction of 2+ points between time 1 and time 2, improvement defined as increase of 2+ between time points, cognitive stability defined as change of < 2 points.	In cognitive decline group, statistically significantly higher incidence of thalamic (11.43 vs. 5.66%, *p* = 0.023) and right sided lesions (6.67 vs. 1.97%, *p* = 0.004). Thalamic infarction increased risk of cognitive decline (OR 2.152, 95% CI 1.095–4.227). Thalamic infarction quadrupled the risk of cognitive decline (OR 4.873, 95% CI 1.634–14.534) in fully adjusted model.
**MRI: DTI, DWI**					
Aben et al. (47)	Ischemic stroke patients with no prior cognitive disorder [*n* = 75, mean age = 70 ±8.5, NIHSS =2 (2–4)]	1 year: 2- time points Cognitive assessments were done at time 1 (baseline 5 weeks ±1 week) and time 2 (after 1 year). MRI was done at time 1 only.	Lesion impact score, calculated by multiplying the percentage of node volume affected by the infarct with the node's corresponding hub-score.	4 cognitive domains: attention and processing speed, working memory and learning, and frontal executive function	- A higher lesion impact score, indicating an increasing infarct size in nodes with a higher hub-score, was related to lower global brain network efficiency [β = −0.528 (−0.776 to −0.277); *P* < 0.001]. - A lower lesion impact score was an independent predictor of cognitive recovery 1 year after stroke [OR = 0.434 (0.193–0.978); *P* = 0.044].
Aben et al. (48)	Ischemic stroke patients (*n* = 217, aged ≥50 years, and MoCA < 26 during hospitalization)	1 year: 2- time points Cognitive assessments were done at time 1 (baseline 5 weeks) and time 2 (after 1 year). MRI was done at time 1 only.	4 DWI-based measures of brain connectivity: global network efficiency and mean connectivity strength, both weighted for MD and FA.	MoCA	- Of 135 patients with PSCI at time 1, 41 (30%) showed cognitive recovery. three out of four DTI measures of brain connectivity: global efficiency FA weighted, mean connectivity strength FA weighted, and mean connectivity strength MD weighted predict cognitive recovery 1 year after IS. These measures, however, did not add a better predictive value over the multivariable model.
Kuceyeski et al. (49)	IS patients (*n* = 40, mean age = 68.1 ± 13.2 years, NIHSS: 6.8 ± 5.6)	6 months Cognitive assessment after discharge and at 6 months. Imaging was done at baseline (within 14 days)	Connectome disruption at three levels: whole brain, individual gray matter regions and between pairs of gray matter regions. Lesion volume model for comparison.	Computer adaptive version of the Activity Measure for Post-Acute Care.	- The regional disconnection model best predicted applied cognitive functioning (*R*^2^ = 0.56) - The pairwise disconnection model best predicted the daily activity measure (*R*^2^ = 0.72)

fMRI, Functional Magnetic Resonance Imaging; FLAIR, Fluid-Attenuated Inversion Recovery; DTI, Diffusion Tensor Imaging; DWI, Diffusion-Weighted Imaging; IS, Ischemic Stroke; rsFC, resting-state functional connectivity; DMN, Default Mode Network; SN, Salience Network; CEN, Central Executive network; MoCA, Montreal Cognitive Assessment; MMSE, Mini-Mental State Examination; PSCI, Post-Stroke Cognitive Impairment; PCC, Posterior Cingulate Cortex; PCu, Precuneus; MPFC, Medial Prefrontal Cortex; FIM, Functional-Independence Measures; WMH, White Matter Hyperintensities; NFL, Neurofilament Lights; ZCT, Zazzo's Cancellation Task; IST, Issac Set a Test; cSS, Cortical Superficial Siderosis; CMI, Cortical Cerebral Microinfarct; MD, Mean Diffusivity; FA, Fractional Anisotropy; GPT, Grooved Pegboard Test.

#### Markers of cerebral small vessel disease

An important study by Sagnier et al. reported that first-time IS patients with pre-existing severe WMH on MRI had less improvement in verbal fluency tests at 3 months to 1 year after IS (38). Similarly, the presence of Cortical Superficial Siderosis (cSS) after first-time IS, another radiologic biomarker of cerebral SVD, was an indicator of worse cognitive recovery in tests of processing speed and attention, independent of IS volume/location, gray matter volume, other SVD biomarkers, and clinical severity, cardiovascular risk factors, and demographic confounders (38).

Similarly, Fruthwirth et al. found that deep WMH volume at baseline predicted recovery of set-shifting at 15 month follow up among patients with recent small subcortical infarcts: participants with no or mild deep WMH improved in set-shifting, while those with moderate to severe WMH showed no improvement (41). A similar pattern was seen for periventricular WMH (pWMH) at baseline and recovery of attention, although this interaction was no longer significant after controlling for age. For set-shifting, mild pWMH predicted improvement, whereas no pWMH and moderate-severe pWMH demonstrated no improvement. Of note, this study found that all patients, regardless of baseline WMH volume, improved in Montreal Cognitive Assessment (MoCA) scores, processing speed, and attention.

Another study examining cerebral small vessel disease (mCSVD) score—determined by MRI evaluation—and medial temporal atrophy (MTA) score—a measure of hippocampal atrophy—found that neither of these imaging markers predicted change in MoCA scores between baseline and 1 year follow up (44). Despite these imaging markers not being associated with a change in cognitive scores, this study did find that mCSVD and MTA scores could be used to predict low vs. high cognitive performance at 1 year, with higher mCSVD and MTA scores associated with increased likelihood of low MoCA scores at follow up.

#### Cortical volume

Sagnier found that total gray matter (GM) volume was the only radiographic factor predictive of cognitive improvement at 12 month follow up among IS patients. Specifically, GM volume of left fronto-temporo-insular regions, right temporo-insular cortex, and basal ganglia were significantly associated with cognitive improvement (43).

#### Infarct characteristic

Several studies examined the impact of infarct location on cognitive recovery using structural MRI. Turunen examined differences in cognitive recovery for cortical vs. subcortical lesions among patients with first-ever supratentorial ischemic stroke (45). While this study found that subcortical infarctions were associated with decreased verbal memory and psychomotor speed in the acute phase and persistent verbal memory differences at 6-month follow-up, there was no difference in recovery of cognition between the two lesion location groups. In contrast, a recent study by Zhang et al. found a significantly higher incidence of thalamic and right-sided lesions in the group that cognitively declined at follow up, determined by a difference in MoCA scores between baseline and 12-month exam (46). In their fully adjusted models, infarct in the thalamus more than quadrupled the risk of cognitive decline among these patients. A recent study further characterized cognitive recovery among patients with ischemic thalamic stroke: in this case control study with a 2-year follow-up period, patients with anterior and inferolateral thalamic strokes were found to have poorer recovery of language, memory, and executive function than those with paramedian strokes (42).

Together, these studies support the use of structural MRI sequences to predict changes in cognition after ischemic stroke. Baseline measures of cerebral SVD, including WMH, as well as baseline cortical volume may be important indicators of the potential for restoration of cognitive ability after stroke. In addition, these articles suggest that different infarct locations as seen on structural MRI may be used to predict differing cognitive recovery trajectories after stroke, although more studies are needed to fully elucidate this relationship.

### Functional magnetic resonance imaging (fMRI)

Only one study reported post-IS cognitive recovery using resting-state fMRI (rs-fMRI) (Table 1) (40). Rs-fMRI utilizes Blood Oxygen Level Dependent (BOLD) to study spontaneous brain neural activity at rest in a specific functional brain region (rsfMRI activity) (50). In their study examining cognitive recovery after IS, Vincentini et al. found weaker interhemispheric spontaneous temporal correlations between different brain functional regions [rs-functional connectivity (rsFC)] within the Default Mode Network (DMN). Alterations of rsFc among IS patients have also been previously reported (51–53). Stroke injury has been shown to disrupt communications between hemispheres and results in both intra-and interhemispheric changes in rsFC (52). Vicentini et al. reported no change in rsFC from the subacute to the chronic phase in IS patients (40). This study did find, however, that better cognitive recovery at 6 months was correlated with rsFC in two networks: DMN and Executive Network (40).

This study argues in favor of the potential of rsFC to predict the course of cognitive changes post-IS and the involvement of DMN in the recovery process, although more studies are required to confirm these findings and further understand brain functional networks involved in the acute to chronic phases of cognitive recovery (20). Furthermore, there are still practical challenges to using rsFMRI in clinical settings, such as availability, time, and the need for interpretation expertise. The lack of technique standardization, including variability in rsfMRI data acquisition, preprocessing, and analytical methods, presents another challenge for clinical applicability (50, 54).

### Diffusion tensor imaging (DTI) and diffusion-weighted imaging (DWI)

While fMRI examines the brain's functional integrity, DTI looks at the brain's white matter structural integrity. Fractional anisotropy (FA) is the most common parameter derived from DTI to assess brain structural connectivity. Other DTI measures are mean diffusivity (MD), radial diffusivity (RD), axial diffusivity (AD), independent of direction, and relative anisotropy.

Important studies using DTI/DWI to study cognitive recovery after IS are summarized in Table 1. A notable study by Aben et al. (47) used diffusion-weighted data after IS to create a lesion impact score that reflected the impact of IS size on the brain's network hubs. The authors demonstrated that a lower lesion impact score was an independent predictor of cognitive recovery 1 year after IS while controlling for WMH and infarct volumes (47). The authors showed that this score could also be calculated using structural MRI sequences (T1, FLAIR), which are routinely ordered as part of a stroke workup and therefore easier to implement in a clinical setting to predict long-term recovery after IS (47). A subsequent study compared DTI measures with a multivariable model, including age, education, and infarct size, and found that three out of four DTI measures of brain connectivity (global efficiency FA weighted, mean connectivity strength FA weighted, and mean connectivity strength MD weighted) predicted cognitive recovery 1 year after IS. These measures, however, did not improve prediction over the multivariable model that included education level and infarct size as significant predictors of cognitive recovery (48).

In a different study using DWI, Kuceyeski et al. (49) compared models of connectome disruption to determine which model best predicted recovery after IS. The authors found that the regional disconnection model, which reflects changes in structural connectivity of gray matter regions (WM tracts connecting brain regions) to the rest of the network, best predicted cognitive recovery. This regional disconnection model was found to be superior to models based on lesion volume and other disconnection models (whole brain and pairwise) (49).

The evidence for DTI and DWI as a means of predicting cognitive recovery after stroke is promising. More research is needed to determine the additional utility of this modality over structural MRI and to improve predictive value.

## Study limitations and future directions

To the best of our knowledge, this review is among the first to focus on neuroimaging biomarkers of cognitive recovery among IS patients. This review is limited by inherent challenges in using keywords to search literature, including a lack of consistent use of terminology to characterize study subject matter. We carefully reviewed the literature to ensure our search was as robust as possible but may have inadvertently missed relevant studies. One key aspect of our study that necessitated the exclusion of multiple otherwise relevant articles was that we focused specifically on the association of neuroimaging with cognitive recovery after stroke; each included study reported on the association between baseline neuroimaging and a change in a cognitive measure over time. Articles that merely reported a cognitive outcome (e.g., post-stroke cognitive impairment vs. no post-stroke cognitive impairment) were therefore excluded from this review of neuroimaging biomarkers for cognitive recovery after IS.

In addition, there are several gaps in the current literature, as discussed below:

1. The best time to study cognitive recovery remains unclear and likely stems from uncertainty in the time frame of expected cognitive recovery post-stroke. In our review, we focused on baseline cognitive assessments done within 6 weeks of stroke to ensure clinical relevance and to adequately compare different articles. However, cognitive recovery studies among chronic stroke patients may help answer this question.
2. The ability to detect cognitive recovery depends largely on the sensitivity of the cognitive test used. The studies included in this review used a variety of different tests—all outlined in Table 1—which may limit our ability to compare them and to generalize their results. Screening tests such as the Montreal Cognitive Assessment (MoCA) and Mini-Mental State Exam (MMSE) are gross measures of cognition and may not capture subtle cognitive dysfunction nor subtypes of cognitive impairment (e.g., left neglect, aphasia). MoCA, for instance, is less sensitive to right-hemispheric lesion-based deficits (55). Adequate studies of cognitive recovery may require more detailed assessments tailored to the setting (e.g., brief baseline exams for inpatients, longer baseline exams for outpatients).
3. To be included in this review, we required at least one repeat measure of cognition. However, it should be noted that having longitudinal imaging is also important to show concordance between neuroimaging and cognitive testing parameters across the recovery course.
4. Some studies—which were excluded from this review—utilized cohorts of both ischemic (IS) and hemorrhagic strokes (HS) to study cognitive recovery. IS and IH have different pathophysiologies and different recovery processes (32, 33). Although the differences in recovery between the two-stroke types are not entirely known (56), future post-stroke cognitive recovery studies should analyze stroke types separately.
5. Only some of the reviewed studies explicitly assessed pre-stroke cognitive status. Pre-stroke cognitive status may influence the recovery process and should be taken into consideration (57). Similarly, not all studies mentioned if only first-time IS patients were included (49). In the future, it would be helpful to standardize screening of pre-stroke cognitive status to better allow comparison between studies of cognitive recovery.
6. Finally, many studies in this review combined neuroimaging with non-neuroimaging tools to improve the prediction of cognitive recovery after IS. For example, Sangier created models that incorporated demographic and clinical factors, including age, sex, education, cardiovascular risk factors, and modified Rankin score (38, 43). Such models are currently used to predict cognitive function after stroke, suggesting their utility in predicting cognitive recovery post-stroke; the SIGNAL2 score and the CHANGE score, both examining the risk of post-stroke cognitive impairment, incorporate age and education into their prediction tools, in addition to imaging variables (58, 59).

## Conclusion

In summary, the current literature on cognitive recovery using neuroimaging as a predictive marker, although small, is promising. No imaging tool is ready for use as an established biomarker yet. Future studies should replicate current findings in larger samples using a consistent methodology.

## Author contributions

MT and VK conducted the literature search and drafted the manuscript in equal contribution. MP reviewed the manuscript for intellectual content. IN conceptualized the project and reviewed the manuscript for intellectual content.
