# Supplementary material for: Neuroimaging biomarkers of cognitive recovery after ischemic stroke
Source: Front Neurol. 2022 Dec 14;13:923942. doi: 10.3389/fneur.2022.923942 (PMC9796574; doi:10.3389/fneur.2022.923942)
Supplement: Supplementary file 1 [file Table_1.docx]

**Supplemental Table 1.** Studies of potential relevance but excluded after full-text review

| **Author and year** | **Study population**  **N, type of stroke, age** | **Duration of study** | **Type of imaging and imaging outcomes examined** | **Cognitive measures** | **Findings** |
| --- | --- | --- | --- | --- | --- |
| **fMRI** | | | | | |
| **Cai, 2021 [60]** | **Inclusion:** first ever ischemic stroke, >18 yo, complete neuropsych testing  **Exclusion:** neuropsych comorbidities, unstable, prestrike dementia  **N = 44 poststroke, 21 normal controls** | Acute state: IQCODE, MMSE, Mini-Cog  3 month follow up: neuropsych + MRIs | **MRI, resting-state fMRI** | IQCODE, MMSE, Mini-Cog  **Post-stroke dementia (PSD) diagnosed:** MMSE (cutoff based on education) or adjusted Mini-Cog <3 | Gray matter was reduced in left cerebellum posterior lobe (P <0.05 compared to PSND) and right superior temporal gyrus in PSD (P <0.05 compared to PSND) and decrease in fractional amplitude of low-frequency fluctuations in right inferior frontal gyrus in PSD (P <0.05 compared to PSND) |
| **Dacosta-Aguayo, Graña et al. 2014a** [61] a | First focal IS patients (n=18, mean age = 63.94 ± 8.26 years, NIHSS ≤1)  Healthy controls with no previous neurologic or psychiatric disease (n=18) | 3 months: 2-time points.  Cognitive assessment was done at time 1 (acute phase, within 72h) and 2 (subacute phase, after 3 months).  fMRI at time 2 only (subacute phase, after 3 months). | Resting-state brain network activity.  18 resting-state networks were examined. | MMSE  MoCA  A battery of neuropsychological tests tapping multiple domains: sustained attention, premotor functions, verbal fluency, language, psychomotor speed, and visual-spatial skills | -Stroke patients had significantly increased brain activity in frontal, frontotemporal, DMN, secondary visual networks and decreased activity in basal ganglia and parietal networks.  -Stroke patients with poor cognitive recovery at 3 months (n=10) had similar brain activity as the whole stroke group.  -Stroke patients with a good cognitive recovery at 3 months (n=8) showed increased brain activity in DMN, frontotemporal networks, and decreased activity in the basal ganglia.  -In patients with poor cognitive recovery, semantic fluency and the trail-making part A tests were negatively correlated with basal ganglia activity and positively correlated with frontal network activity. |
| **Ding, et al.,2014** [62] | First IS patients with no previous cognitive impairment or neurologic disease (n=18, 9 with PSCI and 9 without PSCI, mean age≈64 years).  Healthy cognitively unimpaired controls (n=21) | 3 months: 2-time points  Cognitive assessment was done at time 1 (within 10 days) and 2 (after 3 months).  fMRI was done at time 1 only (within 10 days). | rsFC.  Regions of the DMN. | MMSE, MoCA, Hachinski Ischemic Score, clinical dementia rating, executive dysfunction index, neuropsychiatric inventory, Activities of Daily Living Scale. | Compared to healthy controls, both non-PSCI patients and PSCI patients showed significantly decreased rsFC in PCC/PCu and increased rsFC in the MPFC and left hippocampus.  - Non-PSCI patients showed more significantly increased rsFC in the MPFC and hippocampus than PSCI patients. The rsFC in the PCC/PCu was related to the MoCA score measured at a 10-day follow-up. RsFC in the left hippocampus predicted the MoCA score measured at 3 months follow-up. |
| **Tuladhar et al., 2013** [63] | First IS patients with no previous cognitive impairment or neurologic disease (n=20, mean age = 55.1 ±11.8 years, NIHSS=2 (1-16)  Healthy cognitively unimpaired controls (n=21) | 3 months:  Cognitive assessments done between 6 and 8 weeks after stroke.  fMRI done between 9 and 12 weeks after stroke | rsFC.  Regions of the DMN. | MMSE  California verbal learning test. | Stroke patients performed significantly worse than controls on the delayed recall score in the California verbal learning test and MMSE.  -Decreased rsFC in the left medial temporal lobe, posterior cingulate, and MPFC areas within the DMN and reduced rsFC between these regions in stroke patients compared with controls.  -No significant volumetric differences between stroke patients and controls. |
| **Park, Kim et al. 2014** [64] | First right-hemispheric stroke patients with no previous neuropsychiatric comorbidity (n=24, 7 ischemic and 4 intracerebral hemorrhage patients, mean age=55.7 years)  Age-matched controls (n=11). | 6 months: 3-time points  fMRI and cognitive assessments were both done at time 1 (after 1 month), time 2 (after 3 months), and time 3 (after 6 months). | -Resting-state brain activity  - Regions of the DMN. | -MMSE  -The Seoul-computerized neuropsychological tests: attention, verbal memory, nonverbal memory, and visuomotor coordination tests. | -At 1 month after stroke, patients had decreased DMN connectivity within the PCC, Pcu, medial frontal gyrus, and inferior parietal lobes (p<0.05)  -At 3 months after stroke, connectivity in those DMN regions mostly recovered.  -DMN connectivity of the dorsolateral prefrontal cortex in the contralesional hemisphere was significantly correlated with cognitive recovery. |
| **Pirondini, 2022 [65]** | **Inclusion:** first time stroke with clinical evidence of impairment  **Exclusion:**  **N =** 103 (72 at 2 months and 54 at 12 months) + 19 controls | **12 months:** cognitive assessment at time 1 (1-2 weeks), time 2 (3 months), and time 3 (12 months)  Controls did 2 cognitive assessments 3 months apart | MRI, rs-fMRI | “comprehensive battery of 44 behavioral tests across four behavioral domains language, memory, motor, attention and visual function” | Network temporal property analysis: Stroke patients had longer duration of spatial patterns of brain networks in precentral gyrus and anterior cingulum, and shorter duration in occipital lobe and cerebellum. This was associated with white matter damage in association pathways. Restoration of healthy brain dynamics paralleled recovery of cognition function (but not motor function). |
| **Siegel, Seitzman et al.** 2018 [66] | First stroke patients ischemic or hemorrhagic (n=107, mean age=52.8 years)  Matched controls (n=30) | 1 year: 3-time points  fMRI and cognitive assessments were both done at time 1 (within 2 weeks), time 2 (after 3 months), and time 3 (after 1 year). | RsFC, modularity, small worldness, and global efficacy. | Neuropsychological testing:  Attention: Posner visual orienting task, Mesulam symbol cancellation test, Behavioral Inattention Test Star Cancellation; Memory-Brief Visuospatial Memory Test, Hopkin’s Verbal Learning Test, and spatial span; VISUAL-computerized perimetry. | -The degree of integration within and segregation between networks was significantly reduced at 2 weeks but partially recovered by 3 months and 1 year.  -Network recovery correlated with recovery from language, spatial memory, and attention deficits domains |
| **Ramsey et al., 2016** [67] | First stroke patients (n=77, mean age= 53 ± 10), IS (n=65), HS (n=8), other stroke type (n=4). Matched controls (n=31) | 1 year: 3 time-points  fMRI and cognitive assessments were both done at time 1 (within 2 weeks), time 2 (3 months), and time 3 (1 year). | rsFC across multiple networks. | A battery of neuropsychological testing (memory, attention, language, and visual). | Improvement of attention deficits was correlated with improvement of decreased interhemispheric rsFC, as well as with restoring the normal anti-correlation between dorsal attention/motor regions and DMN /frontoparietal regions, particularly in the damaged hemisphere. |
| **MRI: T1-weighted MRI, T2-weighted MRI, FLAIR** | | | | | |
| **Chander, 2017 [59]** | **Development:** acute ischemic stroke patients, mRS </=2 at discharge, no baseline cog impairment (n=209)  **Internal validation:** same as development (n=185)  **External validation:** STRIDE**,** same as development (n=693) | **Development:** follow up at time 1 (3-6 months after stroke)  **Internal validation:** follow-up time 1 (3-6 months) and time 2 (12-18 months)  **External validation:** follow-up time 1 (3-6 months) and time 2 (12-18 months) | MRI | **Development:** MMSE, MoCA (if needed)  **External validation:** MMSE, MoCA, Clinical Dementia Rating scale, and Geriatric Depression Scale | **Model:** GCA, WMH, non-lacunar cortical infarcts significantly different b/w PSCI and NCI. AUROC 0.82.  **Internal validation:** AUROC 0.78 at 3-6 mo; 0.79 at 12-18 mo  **External validation:** 0.75 at 3-6 mo; 0.74 at 12-18 mo |
| **Chaudhari, 2014 [68]** | Inclusion: Acute ischemic or hemorrhagic stroke,  Exclusion: SAH, dementia, aphasia/impaired consciousness, psychiatric disorders excluded  N=102; n=90 ischemic stroke | Follow up at 3 months and 6months | MRI | **Baseline:** MMSE, short portable mental status questionnaire, clinical eval of cognitive domains  **Follow up:** cognitive assessment repeated  Patients divided into vascular cognitive impairment (VCI) vs. no VCI based on 6 month scores | 45.1% developed VCI.  Strategic site lesion (p < 0.001), higher ARWMC (P = 0.001) associated with VCI at 6 months. No association with laterality or type of stroke (ischemic vs. hemorrhagic). |
| **Clancy, 2022 [69]:** | Inclusion: acute minor stroke  Exclusion: baseline dementia  N= 264 | 3 years:  Cognitive assessment at time 1 (3 months after stroke), time 2 (1 year after stroke) and time 3 (3 years after stroke).  MRI at time 1 and time 2. | MRI at baseline and 1 year | Addebrooke’s Cognitive Examination-Revised (ACE-R) and mRS at all 3 visits. NIHSS at baseline and 1 year. | Cross sectional: increased WMH (at 1 year) associated with decreased cognition at 1 year (P=0.001) but not at three years; WMH not associated with mRS at any time point.  Longitudinal: change in cognition score associated with changed in normalized WMH at baseline and one year (p=0.007)  Multivariate: change in both ACE-R and mRS scores between baseline and 1 year were more likely to have change in the WMH volumes |
| **Hagberg, 2019 [70]** | **Inclusion:** first ever stroke (including hemorrhagic) or TIA  **Exclusion:** SAH, dementia, MCI prior to stroke  **N=**208 at baseline (164 ischemic, 28 TIA, 16 hemorrhagic), 184 1 year follow up, 109 7 year follow up | **7 years:**  Cognitive assessment and MRI at time 1 (baseline), time 2 (12 month), and time 3 (7 years) | **MRI** | MMSE, clock drawing, TMT A/B, 10 word memory.  At 7 year follow up, additionally MoCA and Controlled Oral Word Association Test  Dementia/MCI diagnosed at 1 year and 7 years. | Univariate: on 12 month MRI, lower Fazekas score, lower MTA grade, less GCA associated with favorable cognition at 7 years (all p < 0.000)  Multivariate: lower MTLA grade on MRI at 12 months predicted favorable outcome at 7 years (0.008) |
| **Khan, Heiser et al.** 2019 [71] | IS patients (n=109, mean age=66.6 ± 12.4 years, NIHSS = 7[7-12]). | Retrospective analysis of patients admitted to inpatient rehabilitation. | Infarct volume, leukoaraiosis severity using Fazekas scale  (0-2) for none to mild Leukoaraiosis and (3-6) for moderate to severe. | FIM cognitive subscores on admission and discharge. | Infarct volume (β −0.012, CI −0.019– −0.005; p=0.002) and leukoaraiosis severity (β −0.822, CI −1.223– −0.410; p=0.0001) independently predicted FIM cognitive scores at discharge from Acute inpatient rehabilitation. |
| **Jiang, 2022 [72]** | **Inclusion:** minor ischemic stroke (NIHSS </=3)  **Exclusion**: hemorrhagic stroke, severe comorbidities, white matter lesions with another cause  N = 225 | **2 years**  Cognitive assessment and MRI at time 1 (baseline) and time 2 (2 year follow up) | **MRI –** FLAIR used for WMH volume | Telephone Interview for Cognitive Status (TICS) (telephone adaptation of MMSE)  No CI: TICS-m 21-40; mild CI: TICS-m 13-20; moderate CI: TICS-m <12. | -WMH progression was predicted by lacunes at baseline (antihypertensives protected against progression)  -subjects with WMH progression more likely had cognitive decline (p=0.007)  -cognitive decline associated with baseline CSVD markers (p=0.006), lacunes (p=0.031), CMBs (p=0.006). In adjusted model, only progressive WMH volume associated with cognitive decline (p < 0.001). |
| **Kandiah, 2016 [58]** | **Development:** acute mild ischemic stroke, mRS </=2, no pre-stroke cognitive impairment. PSCI MMSE < 25 or MoCA < 22. N=209.  **(Internal) Validation**: prospective, n=185 | **Development:** cognitive assessment at time 1 (3-6 months from stroke)  **Validation:** cognitive assessment at time 1 (3-6 months) and time 2 (12-18 months after stroke) | MRI and MRA | **Development:** MMSE +/- MoCA  **Validation**: MMSE and MoCA, neuropsychological assessments, depression screen | WMH (b=0.60, p= 0.005), GCA (b=0.40, p=0.147), non-lacunar acute infarcts b=.39, p=0.061, chronic lacunes b=0.58, p = 0.1006, intracranial stenosis b=1.23, p<0.001.  Score uses stenosis, GCA, WMH, lacune count.  Development: AUC 0.829, 73.21% accurate.  Validation: AUC 0.7755 at 3-6 months, AUC 0.783 at 12-18 months |
| **Liang, 2019 [73]** | **Inclusion:** first ever acute ischemic stroke  **Exclusion:** dementia/other neuro disease, severe comorbidities  **N =** 451 | **14 months:** cognitive assessment at time 1 (3 months), time 2 (9 months), time 3 (15 months)  MRI within 7 days of stroke. | MRI within 7 days  SVD score based on WMH, lacunes, cerebral microbleeds, and enlarged perivascular spaces. | MMSE  Cognitive impairment: MMSE </= 26 | Model 1 (adjustment for demographic variables): MMSE scores decreased by 0.36 units per SVD score (standard error = 0.12, *P* = .003.  Model 2 (adjustment for vascular risks, stroke severity, characteristics of acute infarcts, and depressive symptoms): MMSE scores decreased by 0.33 units per SVD score rise (SE = 0.13, *P* = .008)  Model 4: (adjusting for demographic, clinical, and imaging characteristics): WMHs was only significant predictor of cognitive dysfunction (β = −0.24, SE = 0.08, *P* = .001) |
| **Mok, 2012 [74]** | Inclusion: VITATOPS study, RTC evaluating B vitamins for secondary stroke prevention. Ischemic stroke patients with confluent WMH on MRI  Exclusion: hemorrhagic stroke, severe CI  N = 100, 52 in active group. 84 completed 2 year follow up. | Baseline MRI (within 1 week)  Cognitive assessment: Time 1 (3 months), Time 2 (6 months), Time 3 (12 months), Time 4 (18 months), Time 5 (24 months) | MRI within one week | Clinical dementia rating scale (3, 6, 12, 18, 24 months)  MMSE, mattis dementia rating scale (3, 12, 18, 24) | **Change in clinical dementia rating scale**: Predictor of cognitive decline: small cortical gray matter (cGM) volume (p =0.004), small bilateral hippocampal volume (p = 0.061)  Multivariate: cGM volume predicts decline ( p = 0.032 or 0.021 depending on model)  **Change in MMSE/MDRS:** cGM volume only significant predictor of decline in scores in univariate and multivariate |
| **Molad, 2019 [75]** | Inclusion: mild-moderate first ever ischemic stroke or TIA  Exclusion:  N = 397 | 2 years  MRI, cognitive assessment at time 1 (admission/within 7 days).  Cognitive assessment at time 2 (2 year follow up). | MRI – T1 for volume analysis, cortical thickness  DTI | MoCA, NeuroTrax computerized cognitive testing (baseline and 2 years later)  Global cognitive score calculated | **MRI measures that predicted cognitive impairment** (significant after adjustment): Low GM and frontal cortex volume, high WMH volume, and high CSF volume (HR, 2.55; 95% CI, 1.16–5.59, HR, 2.65; 95% CI, 1.15–6.11 and HR, 3.06; 95% CI, 1.45–4.9, respectively). Dose-dependent relationship between the number of MRI markers and the risk of developing PSCI (*p* = 0.001) |
| **Molad, 2017 [76]** | Inclusion: first ever mild to moderate ischemic stroke or TIA    N = 266 (217 with ischemic stroke, 49 with TIA) | 1 year:  MRI at time 1 (within 7 days), cognitive testing at time 2 only (12 month follow up) | MRI within 7 days of stroke  T1 – WMH, enlarged perivascular spaces  T2 - microbleeds | Neurotrax computerized cognitive testing at 12 months following stroke  Cognitive impairment defined by global cognition score at 12 months | WMH significantly associated with cognitive impairment at 12 months (OR 1.517 95% CI 1.006–2.290)  Lacunes count, PVS, and microbleeds did not predict cognitive performance. |
| **O’Sullivan, 2022 [77]** | **Inclusion:** first ischemic stroke, >50 yo, imaging confirmed  **Exclusion:** previous infarct, dementia/CI, aphasia  **N = 51** | Cognitive testing and MRI at time 1 (3 months after stroke) and time 2 (1 year follow up)  MRI at time 1 | MRI | MoCA, free and cued selective reminding task, digit span at 3 months and 1 year | Fornix, hippocampus, and ChBF at baseline (3 months) predicted cognitive improvement at 12 months |
| **Palvovic, 2014 [78]:** | Inclusion: first ever small subcortical, lacunar stroke patients who had evidence of cerebral small vessel disease on imaging  Exclusion:  N = 454, 310 with normal cognition at baseline, 294 with 3-5 year follow up | 3-5 years  Baseline MRI  Time 1 (1-3 months): baseline cognitive assessment  Time 2 (3-5 year follow up): repeat cognitive assessment | MRI  T2 – WMH severity, age related white matter changes  T2/FLAIR – number of lacunar infarcts | MMSE, TMA A/B, Wisconsin card sorting, rey osterreith complex figure, rey auditory verbal learning, Boston naming, animal naming.  Only patients without cognitive impairment at 1-3 month assessment were included; cognitive decline defined at 3 years. | Severity of WMH significantly increased risk for vascular cognitive impairment  -independent predictors of VCI were severity of WMH and total number of lacunes |
| **Ramsey et al., 2017** [79] | First stroke patients (n=132, mean age= 53 ± 10), IS (n=102), HS (n=22). Matched controls (n=31) | 1 year: 3 time-points  MRI and cognitive assessments were both done at time 1 (within 2 weeks), time 2 (3 months), and time 3 (1 year). | Lesion volume and topography. | A battery of neuropsychological testing (memory, attention, language, and visual. | Damage to specific white matter tracts produced poorer recovery for several domains.  These included  - attention (superior longitudinal fasciculus II/III),  - language (posterior arcuate fasciculus), and  - motor (corticospinal tract). |
| **Richard, 2020 [80]** | Ischemic or hemorrhagic (TIA excluded). N=68 initial assessment; N=54 complete protocol. | Three assessments ~1 month part each, including MRI and cognitive assessment at each.  Completed working memory training program over 3-4 weeks. | MRIs (used for brain age prediction) | **Baseline:** MoCA, WASI, verbal learning tests, executive function, more. | **Created model to predict brain age based on morphology,** which tested whether brain age correlated with baseline cognition and cognitive gains after memory training.  No significant results, although trends. |
| **Sivakumar, Riaz et al.** 2017 [81] | TIA /minor stroke with no previous history of dementia (total n=150, ischemic stroke n=91, NIHSS ≤ 3) | 3 months: 4-time points  Cognitive assessment was done time 1 (day 1), time 2 (7 days), time 3 (after 1 month), and time 4 (after 3 months)  Imaging was done at time1 (baseline within 72h), time 2 (at 7 days), time 3 (after 1 month) | DWI  WMH volumes | MoCA | No relationship between acute DWI lesion volume and MoCA score at 30 days.  WMH at baseline predicted persistent cognitive deficits at 30 days |
| **Valdes-Hernandez, 2019 [82]** | Inclusion: mild to moderate ischemic stroke (NIHSS <7)  N = 264 | 12 months:  MRI on presentation  Time 1 (1 month after stroke): cognitive testing  Time 2 (12 months): MRI and cognitive testing | MRI: basal ganglia iron deposits (BGID) and microbleeds (BMB) | National Adult Reading Test (NART) and the Revised Addenbrooke’s Cognitive Examination (ACE-R) | Baseline BGID volumes correlated positively with NART scores at both times (ρ = 0.19, p < 0.01).  Baseline volume of BMBs was only associated with the change in the ACE-R visuospatial scores from 1–3 months to 1 year  Baseline WMH burden predicted verbal fluency and visuospatial abilities scores (B = −0.22, p = 0.001) at 12 months after stroke. |
| **Valdes-Hernandez 2021 [83]:** | Inclusion: lacunar stroke syndrome with MRI confirmed lesion  Exclusion: cortical stroke  N = 118 | 3 years  Presentation: MRI  Time 1 (1-3 months): baseline cognitive assessment  Time 2 (1 year): MRI, cognitive assessment  Time 3 (3 year): cognitive assessment | MRI – DWI, FLAIR, T2/T2 (acquired at presentation and 1 year) | ACE-R and NART at 1-3 months (baseline), 1 year, and 3 years | Stroke in the internal/external capsule/lentiform nucleus vs. centrum semiovale did not predict the general cognitive outcome at 1 or 3 years.  Patients with strokes in the right hemispheric centrum semiovale had a greater increase in ACE-R scores at 1 year than those with left-sided lesions. |
| **Veldsman, 2020 [84]** | CANVAS Study participants – ischemic stroke, >18 yo  Discovery data set: stroke patients, N = 73  Validation data set: 22 patients, same cohort | 1 year:  Time 1 (Baseline): CT/MRI and MoCA  Time 2 (3 months): MRI, cognitive assessment  Time 3 (1 year): MRI, cognitive assessment | MRI – FLAIR, T1 to measure gray matter volume | MoCA at baseline  Extended neuropsychological battery at 3 months and 1 year | Structural covariance of the default mode, dorsal attention, executive control, salience, memory and language-related networks was associated with cognitive performance in the attention, executive function, language, memory and visuospatial domains.  Degeneration of the major structural covariance network (SCN) was associated with cognitive decline in attention, memory and language. |
| **Wang, 2016 [85]** | Inclusion: acute ischemic stroke/TIA  Exclusion:  N = 231 | 28 months  Time 1 (Baseline, within 1 week): CT/MRI  Time 2 (3-6 months): cognitive assessment  Time 3 (28 months): cognitive assessment | CT on all patients on arrival  MRI at baseline (within 1 week of admission)  T1, T2, FLAIR, SWI, DWI  Detecting: cerebral microinfarcts (CMIs) | MoCA (at both time points) | Baseline cortical cerebral micro infarcts (CMIs) were associated with decline in visuospatial functional over 28 months (β=0.5; 95% confidence interval, 0.1–1.0; ***P***=0.008, adjusting for brain atrophy, white matter hyperintensities, lacunes, and microbleeds). |
| **Wyss, Dawson et al.** 2019 [86] | TIA/ or IS (n= 317, mean age=66.4±11.3 years). | 2 years  MRI at baseline and 2 years  Cognitive assessment at 6 months and 2 years. | WMH, CSF volumes.  Combined volume (the sum of WMH and CSF volume normalized by intracranial volume). | MMSE | The association between 2-year follow-up MMSE, adjusted by MMSE at 6 months, and combined WMH and CSF volume was 31% greater than WMH alone, and 11% greater than CSF alone. |
| **Yatawara, 2018 [87]** | Inclusion: acute mild ischemic stroke  Exclusion: prestroke dementia, mRS >2 at discharge, MDD, neurodegenerative disease.  N = 150. | 6 months:  Time 1 (Baseline): MRI on initial presentation    Time 2 (6 month follow up): cognitive assessment | MRI at admission  T2/T1: lacunes, WMH severity  T1: global cortical atrophy | Frontal Assessment Battery, Weschsler Ten Word Delayed Recall, Weschsler Digit Span Forward task, Weschsler animal fluency task.  diagnosis of PSD at 6 months | GCA, WMH associated with decreased executive function and language  The effect of prestroke lesions on cognitive domain disruptions had stronger clinical utility in predicting incident PSD, compared to the effects of stroke-related lesions on cognitive domain impairments |
| **MRI: DTI/DTT** | | | | | |
| **Kern, 2022 [88]** | **Inclusion:** acute ischemic stroke or TIA  **Exclusion:**  **N =** 74 patients (63 had 30 day follow up, 58 had 90 day follow up) | 90 days:  Time 1 (Baseline): MRI  Time 2 (30 days follow up): MoCA or t-MoCA  Time 3 (90 days follow up): T-MoCA | **MRIs at** presentation, at 24 hrs, 5 days, or 30 days  DTI/FLAIR protocol using scan closest to acute presentation | **MoCA/t-MoCA** | Infarct volume associated with MoCA at 30d ( p =0.020) and 90d (p = 0.018). Infarct location not associated with MoCA.  Univariate: WMH volume, global FW, MD, FA initial were all associated with MoCA at 30 days (all P < 0.01)  Univariate: Only global FA (p=0.0038) and global FA tissue (p = 0.0003) associated with MoCA at 90 days  In multivariate, global FW, MD, FA and FA_tissue_ were associated with 90-day T-MoCA (n = 56, p = 0.0034–0.049) |
| **Sagnier, 2022 [89]** | Inclusion: acute supratentorial ischemic stroke, >18 yo  Exclusion: prestroke disability related to neurological disorder, mRS > 1 at baseline | 1 year  Time 1 (24-72 hours): MRI  Time 2 (1 year): cognitive assessment | DTI: Normal-appearing white matter (NAWM) fractional anisotropy (FA) | Cognitive assessment: MoCA, Isaacs set test, Zazzo cancelation task | **Univariate:** NAWM FA associated with 1 year cognition and mRS  **Multivariate:**  **Radiographic model -** association between NAWM FA and cognitive scores ([Table 2](https://www.ahajournals.org/doi/10.1161/STROKEAHA.119.026886#T2)), along with mRS (β=−0.24, *P*=0.005).  **Radiographic-clinical model:** NAWM FA remained independently associated only with the mRS measured 1 year after stroke (β=−0.24, *P*=0.04). |
| **Sagnier, Catheline et al. 2020** [90] | Acute Ischemic supratentorial stroke patients with no previous history of neurologic disorder (n=207, mean age= 66±13 years, median NIHSS=3[2-6]) | 1 year  Cognitive assessment after 1 year  MRI was done at baseline (24-72h). | Normal appearing white matter integrity, FA | -MoCA for global cognition  - IST for executive function  - ZCT for processing speed and attention. | Normal appearing white matter integrity FA was significantly associated with all cognitive scores at one year: MoCA (β=0.23, P=0.001), IST (β=0.27, P <0.001), ZCT completion time (β=−0.34, P<0.001), ZCT number of errors (β=−0.25, P<0.001).  In a subgroup analysis by stroke location, Normal appearing WM integrity FA in widespread WM tracts was associated with the IST score in the right hemispheric strokes. |
| **Zamboni, Griffanti et al. 2019** [91] | Patients with TIA or minor IS (n=566, mean age=66.7 ±14.3 years | Cognitive assessment at 1 month and at 3 months | DTI: FA and MD  WMH | MoCA | WMH volumes and MD/FA were strongly associated with cognitive status in patients ≤80 years (all p < 0.001 for WMH, MD, and FA) but not in those >80 years.  -Lower MoCA scores were associated with frontal WMH in patients ≤80 years but not >80 years. |
| **CT** | | | | | |
| **Mehrabian, 2015 [92]** | **Inclusion:** first-ever ischemic stroke, 50-80 yo  **Exclusion:** hemorrhagic stroke, previous CVA, dementia/CI/severe comorbidities  **N =** 85 (74 at 12 mo). 25 controls. | **12 months**  Time 1 (~5 d, baseline): CT, cognitive testing  Time 2 (1 month): cognitive testing  Time 3 (6 months): cognitive testing  Time 4 (12 months): cognitive testing | CT for hippocampal rating, periventricular changes, and deep WMC | MMSE, TMT A/B, Boston Naming, CERAD neuropsychological battery, geriatric depression, Activities of daily living  (all 4 time points) | Hippocampal atrophy predicted cognitive testing at 12 months (MMSE p = 0.001, IST p = 0.001, IADL p = 0.01, delayed recall p = 0.00, recognition p = 0.005, TMT B p = 0.008, TMT A p =0.007.  Basal ganglia lesions predicted time and language neuropsychological measures at 12 months.  PVCs did not predict scores. |

Abbreviations: fMRI; Functional Magnetic Resonance Imaging, FLAIR; Fluid-Attenuated Inversion Recovery, DTI; Diffusion Tensor Imaging, DWI; Diffusion-Weighted Imaging, IS; Ischemic Stroke, TIA; Transient Ischemic Attack, rsFC; resting-state functional connectivity, DMN; Default Mode Network, NIHSS; The NIH Stroke Scale, MoCA; Montreal Cognitive Assessment, MMSE; Mini-Mental State Examination. PSCI; Post-Stroke Cognitive Impairment, PCC; Posterior Cingulate Cortex, PCu; Precuneus, MPFC; Medial Prefrontal Cortex, FIM; Functional-Independence Measures, WMH; White Matter Hyperintensities, CSF; Cerebraospinal Fluid, ZCT; Zazzo’s Cancellation Task, IST; Issac Set a Test, MD; Mean Diffusivity, FA; Fractional Anisotropy, TMT; Trail Making Test.

**REFERENCES**

[1] R.L. Sacco, S.E. Kasner, J.P. Broderick, L.R. Caplan, J.J. Connors, A. Culebras, M.S. Elkind, M.G. George, A.D. Hamdan, R.T. Higashida, B.L. Hoh, L.S. Janis, C.S. Kase, D.O. Kleindorfer, J.M. Lee, M.E. Moseley, E.D. Peterson, T.N. Turan, A.L. Valderrama, H.V. Vinters, C.o.C.S. American Heart Association Stroke Council, Anesthesia, R. Council on Cardiovascular, Intervention, C. Council on, N. Stroke, E. Council on, Prevention, D. Council on Peripheral Vascular, P.A. Council on Nutrition, and Metabolism, An updated definition of stroke for the 21st century: a statement for healthcare professionals from the American Heart Association/American Stroke Association. Stroke 44 (2013) 2064-89.

[2] P. Mehndiratta, S. Chapman Smith, and B.B. Worrall, Etiologic stroke subtypes: updated definition and efficient workup strategies. Curr Treat Options Cardiovasc Med 17 (2015) 357.

[3] S.S. Virani, A. Alonso, H.J. Aparicio, E.J. Benjamin, M.S. Bittencourt, C.W. Callaway, A.P. Carson, A.M. Chamberlain, S. Cheng, F.N. Delling, M.S.V. Elkind, K.R. Evenson, J.F. Ferguson, D.K. Gupta, S.S. Khan, B.M. Kissela, K.L. Knutson, C.D. Lee, T.T. Lewis, J. Liu, M.S. Loop, P.L. Lutsey, J. Ma, J. Mackey, S.S. Martin, D.B. Matchar, M.E. Mussolino, S.D. Navaneethan, A.M. Perak, G.A. Roth, Z. Samad, G.M. Satou, E.B. Schroeder, S.H. Shah, C.M. Shay, A. Stokes, L.B. VanWagner, N.Y. Wang, C.W. Tsao, E. American Heart Association Council on, C. Prevention Statistics, and S. Stroke Statistics, Heart Disease and Stroke Statistics-2021 Update: A Report From the American Heart Association. Circulation 143 (2021) e254-e743.

[4] L.B. Ovbiagele B Fau - Goldstein, R.T. Goldstein Lb Fau - Higashida, V.J. Higashida Rt Fau - Howard, S.C. Howard Vj Fau - Johnston, O.A. Johnston Sc Fau - Khavjou, D.T. Khavjou Oa Fau - Lackland, J.H. Lackland Dt Fau - Lichtman, S. Lichtman Jh Fau - Mohl, R.L. Mohl S Fau - Sacco, J.L. Sacco Rl Fau - Saver, J.G. Saver Jl Fau - Trogdon, and J.G. Trogdon, Forecasting the future of stroke in the United States: a policy statement from the American Heart Association and American Stroke Association. (2013).

[5] E.J. Benjamin, P. Muntner, A. Alonso, M.S. Bittencourt, C.W. Callaway, A.P. Carson, A.M. Chamberlain, A.R. Chang, S. Cheng, S.R. Das, F.N. Delling, L. Djousse, M.S.V. Elkind, J.F. Ferguson, M. Fornage, L.C. Jordan, S.S. Khan, B.M. Kissela, K.L. Knutson, T.W. Kwan, D.T. Lackland, T.T. Lewis, J.H. Lichtman, C.T. Longenecker, M.S. Loop, P.L. Lutsey, S.S. Martin, K. Matsushita, A.E. Moran, M.E. Mussolino, M. O'Flaherty, A. Pandey, A.M. Perak, W.D. Rosamond, G.A. Roth, U.K.A. Sampson, G.M. Satou, E.B. Schroeder, S.H. Shah, N.L. Spartano, A. Stokes, D.L. Tirschwell, C.W. Tsao, M.P. Turakhia, L.B. VanWagner, J.T. Wilkins, S.S. Wong, S.S. Virani, E. American Heart Association Council on, C. Prevention Statistics, and S. Stroke Statistics, Heart Disease and Stroke Statistics-2019 Update: A Report From the American Heart Association. Circulation 139 (2019) e56-e528.

[6] L.A. Boyd, K.S. Hayward, N.S. Ward, C.M. Stinear, C. Rosso, R.J. Fisher, A.R. Carter, A.P. Leff, D.A. Copland, L.M. Carey, L.G. Cohen, D.M. Basso, J.M. Maguire, and S.C. Cramer, Biomarkers of Stroke Recovery: Consensus-Based Core Recommendations from the Stroke Recovery and Rehabilitation Roundtable. Neurorehabil Neural Repair 31 (2017) 864-876.

[7] J. Bernhardt, K. Borschmann, L. Boyd, S.T. Carmichael, D. Corbett, S.C. Cramer, T. Hoffmann, G. Kwakkel, S. Savitz, G. Saposnik, M. Walker, and N. Ward, Moving Rehabilitation Research Forward: Developing Consensus Statements for Rehabilitation and Recovery Research. Neurorehabil Neural Repair 31 (2017) 694-698.

[8] G. Hartwigsen, and D. Saur, Neuroimaging of stroke recovery from aphasia - Insights into plasticity of the human language network. Neuroimage 190 (2019) 14-31.

[9] M.R.T. Sinke, G.A.F. van Tilborg, A.E. Meerwaldt, C.L. van Heijningen, A. van der Toorn, M. Straathof, F. Rakib, M.H.M. Ali, K. Al-Saad, W.M. Otte, and R.M. Dijkhuizen, Remote Corticospinal Tract Degeneration After Cortical Stroke in Rats May Not Preclude Spontaneous Sensorimotor Recovery. Neurorehabil Neural Repair (2021) 15459683211041318.

[10] D. Mattia, F. Pichiorri, E. Colamarino, M. Masciullo, G. Morone, J. Toppi, I. Pisotta, F. Tamburella, M. Lorusso, S. Paolucci, M. Puopolo, F. Cincotti, and M. Molinari, The Promotoer, a brain-computer interface-assisted intervention to promote upper limb functional motor recovery after stroke: a study protocol for a randomized controlled trial to test early and long-term efficacy and to identify determinants of response. BMC Neurol 20 (2020) 254.

[11] G. Liu, S. Tan, K. Peng, C. Dang, S. Xing, C. Xie, and J. Zeng, Network change in the ipsilesional cerebellum is correlated with motor recovery following unilateral pontine infarction. Eur J Neurol 26 (2019) 1266-1273.

[12] W. Wei, L. Bai, J. Wang, R. Dai, R.K. Tong, Y. Zhang, Z. Song, W. Jiang, C. Shi, M. Li, L. Ai, and J. Tian, A longitudinal study of hand motor recovery after sub-acute stroke: a study combined FMRI with diffusion tensor imaging. PLoS One 8 (2013) e64154.

[13] C. Dang, G. Liu, S. Xing, C. Xie, K. Peng, C. Li, J. Li, J. Zhang, L. Chen, Z. Pei, and J. Zeng, Longitudinal cortical volume changes correlate with motor recovery in patients after acute local subcortical infarction. Stroke 44 (2013) 2795-801.

[14] E. Zarahn, L. Alon, S.L. Ryan, R.M. Lazar, M.S. Vry, C. Weiller, R.S. Marshall, and J.W. Krakauer, Prediction of motor recovery using initial impairment and fMRI 48 h poststroke. Cereb Cortex 21 (2011) 2712-21.

[15] M. Iorga, J. Higgins, D. Caplan, R. Zinbarg, S. Kiran, C.K. Thompson, B. Rapp, and T.B. Parrish, Predicting language recovery in post-stroke aphasia using behavior and functional MRI. Sci Rep 11 (2021) 8419.

[16] R. Sebastian, C. Long, J.J. Purcell, A.V. Faria, M. Lindquist, S. Jarso, D. Race, C. Davis, J. Posner, A. Wright, and A.E. Hillis, Imaging network level language recovery after left PCA stroke. Restor Neurol Neurosci 34 (2016) 473-89.

[17] G. Hartwigsen, Adaptive Plasticity in the Healthy Language Network: Implications for Language Recovery after Stroke. Neural Plast 2016 (2016) 9674790.

[18] S. Jarso, M. Li, A. Faria, C. Davis, R. Leigh, R. Sebastian, K. Tsapkini, S. Mori, and A.E. Hillis, Distinct mechanisms and timing of language recovery after stroke. Cogn Neuropsychol 30 (2013) 454-75.

[19] K. Lidzba, M. Staudt, F. Zieske, E. Schwilling, and H. Ackermann, Prestroke/poststroke fMRI in aphasia: perilesional hemodynamic activation and language recovery. Neurology 78 (2012) 289-91.

[20] A. Baldassarre, L.E. Ramsey, J.S. Siegel, G.L. Shulman, and M. Corbetta, Brain connectivity and neurological disorders after stroke. Curr Opin Neurol 29 (2016) 706-713.

[21] E.A.-O. Sexton, A. McLoughlin, D.J. Williams, N.A. Merriman, N. Donnelly, D.A.-O. Rohde, A.A.-O. Hickey, M.A. Wren, and K. Bennett, Systematic review and meta-analysis of the prevalence of cognitive impairment no dementia in the first year post-stroke. (2019).

[22] J.H. Sun, L. Tan, and J.T. Yu, Post-stroke cognitive impairment: epidemiology, mechanisms and management. Ann Transl Med 2 (2014) 80.

[23] S. Melkas, H. Jokinen, M. Hietanen, and T. Erkinjuntti, Poststroke cognitive impairment and dementia: prevalence, diagnosis, and treatment. Degener Neurol Neuromuscul Dis 4 (2014) 21-27.

[24] C. Ballard, S. Rowan E Fau - Stephens, R. Stephens S Fau - Kalaria, R.A. Kalaria R Fau - Kenny, and R.A. Kenny, Prospective follow-up study between 3 and 15 months after stroke: improvements and decline in cognitive function among dementia-free stroke survivors >75 years of age. (2003).

[25] T.G. Liman, M. Heuschmann Pu Fau - Endres, A. Endres M Fau - Flöel, S. Flöel A Fau - Schwab, P.L. Schwab S Fau - Kolominsky-Rabas, and P.L. Kolominsky-Rabas, Changes in cognitive function over 3 years after first-ever stroke and predictors of cognitive impairment and long-term cognitive stability: the Erlangen Stroke Project. (2011).

[26] D.W. Desmond, J.T. Moroney, M. Sano, and Y. Stern, Recovery of Cognitive Function After Stroke. Stroke 27 (1996) 1798-1803.

[27] M. Obaid, C. Flach, I. Marshall, D.A.W. C, and A. Douiri, Long-Term Outcomes in Stroke Patients with Cognitive Impairment: A Population-Based Study. LID - 10.3390/geriatrics5020032 [doi] LID - 32. (2020).

[28] B. Casolla, F. Caparros, C. Cordonnier, S. Bombois, H. Hénon, R. Bordet, F. Orzi, and D. Leys, Biological and imaging predictors of cognitive impairment after stroke: a systematic review. J Neurol 266 (2019) 2593-2604.

[29] B.L. Edlow, J. Claassen, N.D. Schiff, and D.M. Greer, Recovery from disorders of consciousness: mechanisms, prognosis and emerging therapies. Nature Reviews Neurology 17 (2021) 135-156.

[30] S. Silva, F. de Pasquale, C. Vuillaume, B. Riu, I. Loubinoux, T. Geeraerts, T. Seguin, V. Bounes, O. Fourcade, J.F. Demonet, and P. Péran, Disruption of posteromedial large-scale neural communication predicts recovery from coma. (2015).

[31] J. Soch, A. Richter, H. Schütze, J.M. Kizilirmak, A. Assmann, G. Behnisch, H. Feldhoff, L. Fischer, J. Heil, L. Knopf, C. Merkel, M. Raschick, C.-J. Schietke, A. Schult, C.I. Seidenbecher, R. Yakupov, G. Ziegler, J. Wiltfang, E. Düzel, and B.H. Schott, A comprehensive score reflecting memory-related fMRI activations and deactivations as potential biomarker for neurocognitive aging. Human Brain Mapping 42 (2021) 4478-4496.

[32] A. Bhalla, Y. Wang, A. Rudd, and C.D. Wolfe, Differences in outcome and predictors between ischemic and intracerebral hemorrhage: the South London Stroke Register. Stroke 44 (2013) 2174-81.

[33] J.W. Wei, J.-G. Heeley El Fau - Wang, Y. Wang Jg Fau - Huang, L.K.S. Huang Y Fau - Wong, Z. Wong Lk Fau - Li, S. Li Z Fau - Heritier, H. Heritier S Fau - Arima, C.S. Arima H Fau - Anderson, and C.S. Anderson, Comparison of recovery patterns and prognostic indicators for ischemic and hemorrhagic stroke in China: the ChinaQUEST (QUality Evaluation of Stroke Care and Treatment) Registry study. (2010).

[34] A.G. Sorensen, and H. Ay, Transient ischemic attack: definition, diagnosis, and risk stratification. Neuroimaging Clin N Am 21 (2011) 303-x.

[35] A.L. Abbott, M. Silvestrini, R. Topakian, J. Golledge, A.M. Brunser, G.J. de Borst, R.E. Harbaugh, F.N. Doubal, T. Rundek, A. Thapar, A.H. Davies, A. Kam, and J.M. Wardlaw, Optimizing the Definitions of Stroke, Transient Ischemic Attack, and Infarction for Research and Application in Clinical Practice. Frontiers in neurology 8 (2017) 537-537.

[36] R.F. Gottesman, and A.E. Hillis, Predictors and assessment of cognitive dysfunction resulting from ischaemic stroke. Lancet Neurol 9 (2010) 895-905.

[37] Y. Peng, Q. Li, L. Qin, Y. He, X. Luo, Y. Lan, X. Chen, X. Wang, and Q.M. Wang, Combination of Serum Neurofilament Light Chain Levels and MRI Markers to Predict Cognitive Function in Ischemic Stroke. Neurorehabil Neural Repair 35 (2021) 247-255.

[38] S. Sagnier, G. Catheline, F. Munsch, A. Bigourdan, M. Poli, S. Debruxelles, P. Renou, S. Olindo, F. Rouanet, V. Dousset, T. Tourdias, and I. Sibon, Severity of Small Vessel Disease Biomarkers Reduces the Magnitude of Cognitive Recovery after Ischemic Stroke. Cerebrovasc Dis 50 (2021) 456-463.

[39] S. Sagnier, G. Okubo, G. Catheline, F. Munsch, A. Bigourdan, S. Debruxelles, M. Poli, S. Olindo, P. Renou, F. Rouanet, V. Dousset, T. Tourdias, and I. Sibon, Chronic Cortical Cerebral Microinfarcts Slow Down Cognitive Recovery After Acute Ischemic Stroke. Stroke 50 (2019) 1430-1436.

[40] V. Fruhwirth, C. Enzinger, S. Fandler-Höfler, M. Kneihsl, S. Eppinger, S. Ropele, R. Schmidt, T. Gattringer, and D. Pinter, Baseline white matter hyperintensities affect the course of cognitive function after small vessel disease-related stroke: a prospective observational study. Eur J Neurol 28 (2021) 401-410.

[41] P.S. Sung, K.P. Lee, P.Y. Lin, H.C. Su, R.L. Yu, K.J. Tsai, S.H. Lin, and C.H. Chen, Factors Associated with Cognitive Outcomes After First-Ever Ischemic Stroke: The Impact of Small Vessel Disease Burden and Neurodegeneration. J Alzheimers Dis 83 (2021) 569-579.

[42] S. Sagnier, G. Catheline, B. Dilharreguy, F. Munsch, A. Bigourdan, M. Poli, S. Debruxelles, S. Olindo, P. Renou, F. Rouanet, V. Dousset, T. Tourdias, and I. Sibon, Admission Brain Cortical Volume: An Independent Determinant of Poststroke Cognitive Vulnerability. Stroke 48 (2017) 2113-2120.

[43] K.E. Turunen, T.V. Kauranen, S.P. Laari, S.M. Mustanoja, T. Tatlisumak, and E.T. Poutiainen, Cognitive deficits after subcortical infarction are comparable with deficits after cortical infarction. Eur J Neurol 20 (2013) 286-92.

[44] C. Zhang, Y. Wang, S. Li, Y. Pan, M. Wang, X. Liao, J. Shi, and Y. Wang, Infarct location and cognitive change in patients after acute ischemic stroke: The ICONS study. J Neurol Sci 438 (2022) 120276.

[45] A.C. Scharf, J. Gronewold, O. Todica, C. Moenninghoff, T.R. Doeppner, B. de Haan, C.L.A. Bassetti, and D.M. Hermann, Evolution of Neuropsychological Deficits in First-Ever Isolated Ischemic Thalamic Stroke and Their Association With Stroke Topography: A Case-Control Study. Stroke 53 (2022) 1904-1914.

[46] J.E. Vicentini, M. Weiler, R.F. Casseb, S.R. Almeida, L. Valler, B.M. de Campos, and L.M. Li, Subacute functional connectivity correlates with cognitive recovery six months after stroke. Neuroimage Clin 29 (2021) 102538.

[47] H. Lv, Z. Wang, E. Tong, L.M. Williams, G. Zaharchuk, M. Zeineh, A.N. Goldstein-Piekarski, T.M. Ball, C. Liao, and M. Wintermark, Resting-State Functional MRI: Everything That Nonexperts Have Always Wanted to Know. AJNR Am J Neuroradiol 39 (2018) 1390-1399.

[48] Y. Zhu, L. Bai, P. Liang, S. Kang, H. Gao, and H. Yang, Disrupted brain connectivity networks in acute ischemic stroke patients. Brain Imaging and Behavior 11 (2017) 444-453.

[49] J.S. Siegel, L.E. Ramsey, A.Z. Snyder, N.V. Metcalf, R.V. Chacko, K. Weinberger, A. Baldassarre, C.D. Hacker, G.L. Shulman, and M. Corbetta, Disruptions of network connectivity predict impairment in multiple behavioral domains after stroke. Proc Natl Acad Sci U S A 113 (2016) E4367-76.

[50] C. Tang, Z. Zhao, C. Chen, X. Zheng, F. Sun, X. Zhang, J. Tian, M. Fan, Y. Wu, and J. Jia, Decreased Functional Connectivity of Homotopic Brain Regions in Chronic Stroke Patients: A Resting State fMRI Study. PloS one 11 (2016) e0152875.

[51] C. Hohenfeld, C.J. Werner, and K. Reetz, Resting-state connectivity in neurodegenerative disorders: Is there potential for an imaging biomarker? Neuroimage Clin 18 (2018) 849-870.

[52] H.P. Aben, G.J. Biessels, N.A. Weaver, J.M. Spikman, J.M.A. Visser-Meily, P.L.M. de Kort, and Y.D. Reijmer, Extent to Which Network Hubs Are Affected by Ischemic Stroke Predicts Cognitive Recovery. Stroke 50 (2019) 2768-2774.

[53] H.P. Aben, L. De Munter, Y.D. Reijmer, J.M. Spikman, J.M.A. Visser-Meily, G.J. Biessels, and P.L.M. De Kort, Prediction of Cognitive Recovery After Stroke: The Value of Diffusion-Weighted Imaging-Based Measures of Brain Connectivity. (2021).

[54] A. Kuceyeski, B.B. Navi, H. Kamel, A. Raj, N. Relkin, J. Toglia, C. Iadecola, and M. O'Dell, Structural connectome disruption at baseline predicts 6-months post-stroke outcome. Hum Brain Mapp 37 (2016) 2587-601.

[55] E. Chan, S. Altendorff, C. Healy, D.J. Werring, and L. Cipolotti, The test accuracy of the Montreal Cognitive Assessment (MoCA) by stroke lateralisation. Journal of the Neurological Sciences 373 (2017) 100-104.

[56] R. Perna, and J. Temple, Rehabilitation Outcomes: Ischemic versus Hemorrhagic Strokes. Behavioural Neurology 2015 (2015) 891651.

[57] C. Reitz, M.J. Bos, A. Hofman, P.J. Koudstaal, and M.M.B. Breteler, Prestroke Cognitive Performance, Incident Stroke, and Risk of Dementia. Stroke 39 (2008) 36-41.

[58] N. Kandiah, R.J. Chander, X. Lin, A. Ng, Y.Y. Poh, C.Y. Cheong, A.R. Cenina, and P.N. Assam, Cognitive Impairment after Mild Stroke: Development and Validation of the SIGNAL2 Risk Score. J Alzheimers Dis 49 (2016) 1169-77.

[59] R.J. Chander, B.Y.K. Lam, X. Lin, A.Y.T. Ng, A.P.L. Wong, V.C.T. Mok, and N. Kandiah, Development and validation of a risk score (CHANGE) for cognitive impairment after ischemic stroke. Sci Rep 7 (2017) 12441.

[60] H. Cai, Z. Zhao, L. Ni, G. Han, X. Hu, D. Wu, X. Ding, and J. Wang, Structural and Functional Deficits in Patients with Poststroke Dementia: A Multimodal MRI Study. Neural Plast 2021 (2021) 3536234.

[61] R. Dacosta-Aguayo, M. Graña, A. Savio, M. Fernández-Andújar, M. Millán, E. López-Cancio, C. Cáceres, N. Bargalló, C. Garrido, M. Barrios, I.C. Clemente, M. Hernández, J. Munuera, A. Dávalos, T. Auer, and M. Mataró, Prognostic value of changes in resting-state functional connectivity patterns in cognitive recovery after stroke: A 3T fMRI pilot study. Hum Brain Mapp 35 (2014) 3819-31.

[62] X. Ding, C.Y. Li, Q.S. Wang, F.Z. Du, Z.W. Ke, F. Peng, J. Wang, and L. Chen, Patterns in default-mode network connectivity for determining outcomes in cognitive function in acute stroke patients. (2014).

[63] A.M. Tuladhar, E. Snaphaan L Fau - Shumskaya, M. Shumskaya E Fau - Rijpkema, G. Rijpkema M Fau - Fernandez, D.G. Fernandez G Fau - Norris, F.-E. Norris Dg Fau - de Leeuw, and F.E. de Leeuw, Default Mode Network Connectivity in Stroke Patients. (2013).

[64] J.Y. Park, Y.H. Kim, W.H. Chang, C.H. Park, Y.I. Shin, S.T. Kim, and A. Pascual-Leone, Significance of longitudinal changes in the default-mode network for cognitive recovery after stroke. Eur J Neurosci 40 (2014) 2715-22.

[65] E. Pirondini, N. Kinany, C.L. Sueur, J.C. Griffis, G.L. Shulman, M. Corbetta, and D. Van De Ville, Post-stroke reorganization of transient brain activity characterizes deficits and recovery of cognitive functions. Neuroimage 255 (2022) 119201.

[66] J.S. Siegel, B.A. Seitzman, L.E. Ramsey, M. Ortega, E.M. Gordon, N.U.F. Dosenbach, S.E. Petersen, G.L. Shulman, and M. Corbetta, Re-emergence of modular brain networks in stroke recovery. Cortex 101 (2018) 44-59.

[67] L.E. Ramsey, J.S. Siegel, A. Baldassarre, N.V. Metcalf, K. Zinn, G.L. Shulman, and M. Corbetta, Normalization of network connectivity in hemispatial neglect recovery. Ann Neurol 80 (2016) 127-41.

[68] T.S. Chaudhari, R. Verma, R.K. Garg, M.K. Singh, H.S. Malhotra, and P.K. Sharma, Clinico-radiological predictors of vascular cognitive impairment (VCI) in patients with stroke: a prospective observational study. J Neurol Sci 340 (2014) 150-8.

[69] U. Clancy, S.D.J. Makin, C.A. McHutchison, V. Cvoro, F.M. Chappell, M. Hernández, E. Sakka, F. Doubal, and J.M. Wardlaw, Impact of Small Vessel Disease Progression on Long-term Cognitive and Functional Changes After Stroke. Neurology 98 (2022) e1459-e1469.

[70] G. Hagberg, B. Fure, B. Thommessen, H. Ihle-Hansen, A.R. Øksengård, S. Nygård, S.T. Pendlebury, M.K. Beyer, T.B. Wyller, and H. Ihle-Hansen, Predictors for Favorable Cognitive Outcome Post-Stroke: A-Seven-Year Follow-Up Study. Dement Geriatr Cogn Disord 48 (2019) 45-55.

[71] M. Khan, H. Heiser, N. Bernicchi, L. Packard, J.L. Parker, M.A. Edwardson, B. Silver, K.V. Elisevich, and N. Henninger, Leukoaraiosis Predicts Short-term Cognitive But not Motor Recovery in Ischemic Stroke Patients During Rehabilitation. J Stroke Cerebrovasc Dis 28 (2019) 1597-1603.

[72] J. Jiang, K. Yao, X. Huang, Y. Zhang, F. Shen, and S. Weng, Longitudinal white matter hyperintensity changes and cognitive decline in patients with minor stroke. Aging Clin Exp Res 34 (2022) 1047-1054.

[73] Y. Liang, Y.K. Chen, Y.L. Liu, V.C.T. Mok, G.S. Ungvari, W.C.W. Chu, S.W. Seo, and W.K. Tang, Cerebral Small Vessel Disease Burden Is Associated With Accelerated Poststroke Cognitive Decline: A 1-Year Follow-Up Study. J Geriatr Psychiatry Neurol 32 (2019) 336-343.

[74] V. Mok, Y. Xiong, K.K. Wong, A. Wong, R. Schmidt, W.W. Chu, X. Hu, E.Y. Lung Leung, S. Chen, Y. Chen, W.K. Tang, X. Chen, C.L. Ho, K.S. Wong, and S.T. Wong, Predictors for cognitive decline in patients with confluent white matter hyperintensities. Alzheimers Dement 8 (2012) S96-s103.

[75] J. Molad, H. Hallevi, A.D. Korczyn, E. Kliper, E. Auriel, N.M. Bornstein, and E. Ben Assayag, Vascular and Neurodegenerative Markers for the Prediction of Post-Stroke Cognitive Impairment: Results from the TABASCO Study. J Alzheimers Dis 70 (2019) 889-898.

[76] J. Molad, E. Kliper, A.D. Korczyn, E. Ben Assayag, D. Ben Bashat, S. Shenhar-Tsarfaty, O. Aizenstein, L. Shopin, N.M. Bornstein, and E. Auriel, Only White Matter Hyperintensities Predicts Post-Stroke Cognitive Performances Among Cerebral Small Vessel Disease Markers: Results from the TABASCO Study. J Alzheimers Dis 56 (2017) 1293-1299.

[77] M.J. O'Sullivan, L.K.L. Oestreich, P. Wright, and A.N. Clarkson, Cholinergic and hippocampal systems facilitate cross-domain cognitive recovery after stroke. Brain 145 (2022) 1698-1710.

[78] A.M. Pavlovic, T. Pekmezovic, G. Tomic, J.Z. Trajkovic, and N. Sternic, Baseline predictors of cognitive decline in patients with cerebral small vessel disease. J Alzheimers Dis 42 Suppl 3 (2014) S37-43.

[79] L.E. Ramsey, J.S. Siegel, C.E. Lang, M. Strube, G.L. Shulman, and M. Corbetta, Behavioural clusters and predictors of performance during recovery from stroke. Nat Hum Behav 1 (2017).

[80] G. Richard, K. Kolskår, K.M. Ulrichsen, T. Kaufmann, D. Alnæs, A.M. Sanders, E.S. Dørum, J. Monereo Sánchez, A. Petersen, H. Ihle-Hansen, J.E. Nordvik, and L.T. Westlye, Brain age prediction in stroke patients: Highly reliable but limited sensitivity to cognitive performance and response to cognitive training. Neuroimage Clin 25 (2020) 102159.

[81] L. Sivakumar, P. Riaz, M. Kate, T. Jeerakathil, C. Beaulieu, B. Buck, R. Camicioli, and K. Butcher, White matter hyperintensity volume predicts persistent cognitive impairment in transient ischemic attack and minor stroke. Int J Stroke 12 (2017) 264-272.

[82] M.D.C. Valdés Hernández, T. Case, F.M. Chappell, A. Glatz, S. Makin, F. Doubal, and J.M. Wardlaw, Association between Striatal Brain Iron Deposition, Microbleeds and Cognition 1 Year After a Minor Ischaemic Stroke. Int J Mol Sci 20 (2019).

[83] M.D.C. Valdés Hernández, T. Grimsley-Moore, F.M. Chappell, M.J. Thrippleton, P.A. Armitage, E. Sakka, S. Makin, and J.M. Wardlaw, Post-stroke Cognition at 1 and 3 Years Is Influenced by the Location of White Matter Hyperintensities in Patients With Lacunar Stroke. Front Neurol 12 (2021) 634460.

[84] M. Veldsman, H.J. Cheng, F. Ji, E. Werden, M.S. Khlif, K.K. Ng, J.K.W. Lim, X. Qian, H. Yu, J.H. Zhou, and A. Brodtmann, Degeneration of structural brain networks is associated with cognitive decline after ischaemic stroke. Brain Commun 2 (2020) fcaa155.

[85] Z. Wang, S.J. van Veluw, A. Wong, W. Liu, L. Shi, J. Yang, Y. Xiong, A. Lau, G.J. Biessels, and V.C. Mok, Risk Factors and Cognitive Relevance of Cortical Cerebral Microinfarcts in Patients With Ischemic Stroke or Transient Ischemic Attack. Stroke 47 (2016) 2450-5.

[86] A. Wyss, J. Dawson, F. Arba, J.M. Wardlaw, and D.A. Dickie, Combining Neurovascular and Neurodegenerative Magnetic Resonance Imaging Measures in Stroke. Stroke 50 (2019) 1136-1139.

[87] C. Yatawara, K.P. Ng, R. Chander, and N. Kandiah, Associations between lesions and domain-specific cognitive decline in poststroke dementia. Neurology 91 (2018) e45-e54.

[88] K.C. Kern, C.B. Wright, and R. Leigh, Global changes in diffusion tensor imaging during acute ischemic stroke and post-stroke cognitive performance. J Cereb Blood Flow Metab 42 (2022) 1854-1866.

[89] S. Sagnier, G. Catheline, B. Dilharreguy, P.A. Linck, P. Coupé, F. Munsch, A. Bigourdan, M. Poli, S. Debruxelles, P. Renou, S. Olindo, F. Rouanet, V. Dousset, T. Tourdias, and I. Sibon, Normal-Appearing White Matter Deteriorates over the Year After an Ischemic Stroke and Is Associated with Global Cognition. Transl Stroke Res 13 (2022) 716-724.

[90] S. Sagnier, G. Catheline, B. Dilharreguy, P.A. Linck, P. Coupé, F. Munsch, A. Bigourdan, S. Debruxelles, M. Poli, S. Olindo, P. Renou, F. Rouanet, V. Dousset, S. Berthoz, T. Tourdias, and I. Sibon, Normal-Appearing White Matter Integrity Is a Predictor of Outcome After Ischemic Stroke. Stroke 51 (2020) 449-456.

[91] G. Zamboni, L. Griffanti, S. Mazzucco, S.T. Pendlebury, and P.M. Rothwell, Age-dependent association of white matter abnormality with cognition after TIA or minor stroke. Neurology 93 (2019) e272-e282.

[92] S. Mehrabian, M. Raycheva, N. Petrova, A. Janyan, M. Petrova, and L. Traykov, Neuropsychological and neuroimaging markers in prediction of cognitive impairment after ischemic stroke: a prospective follow-up study. Neuropsychiatr Dis Treat 11 (2015) 2711-9.
